# Equilibrium and non‐equilibrium dynamics simultaneously operate in the Galápagos islands

**DOI:** 10.1111/ele.12461

**Published:** 2015-06-23

**Authors:** Luis M. Valente, Albert B. Phillimore, Rampal S. Etienne

**Affiliations:** ^1^ Unit of Evolutionary Biology/Systematic ZoologyInstitute of Biochemistry and BiologyUniversity of PotsdamKarl‐Liebknecht‐Strasse 24‐25, Haus 26D‐14476PotsdamGermany; ^2^ Institute of Evolutionary BiologyUniversity of EdinburghEdinburghEH9 3JTUK; ^3^ Groningen Institute for Evolutionary Life SciencesUniversity of GroningenPO Box 11103Groningen9700 CCThe Netherlands

**Keywords:** Community assembly, diversification, dynamic equilibrium, island biogeography, phylogeny

## Abstract

Island biotas emerge from the interplay between colonisation, speciation and extinction and are often the scene of spectacular adaptive radiations. A common assumption is that insular diversity is at a dynamic equilibrium, but for remote islands, such as Hawaii or Galápagos, this idea remains untested. Here, we reconstruct the temporal accumulation of terrestrial bird species of the Galápagos using a novel phylogenetic method that estimates rates of biota assembly for an entire community. We show that species richness on the archipelago is in an ascending phase and does not tend towards equilibrium. The majority of the avifauna diversifies at a slow rate, without detectable ecological limits. However, Darwin's finches form an exception: they rapidly reach a carrying capacity and subsequently follow a coalescent‐like diversification process. Together, these results suggest that avian diversity of remote islands is rising, and challenge the mutual exclusivity of the non‐equilibrium and equilibrium ecological paradigms.

## Introduction

Islands have been prominent in the development of ecological and evolutionary theory, inspiring significant advances to our understanding of global scale biodiversity patterns (Warren *et al*. 2015). MacArthur and Wilson's landmark island biogeography theory presented a model for how biotas assemble through immigration and extinction, ultimately reaching a dynamic equilibrium in the number of species (MacArthur & Wilson 1963, 1967). Whether communities are at or tend towards diversity equilibrium remains a central problem in ecology and evolution (Ricklefs & Bermingham 2001; Rabosky & Glor 2010; Etienne *et al*. 2012; Manceau *et al*. 2015). For remote archipelagos, such as Hawaii or Galápagos, which host spectacular *in situ* radiations and are limited by immigration (Gillespie 2004; Gillespie & Baldwin 2010; Rosindell & Phillimore 2011), the applicability of equilibrium dynamics has been questioned on the basis that diversity perturbations via geological or meteorological events may be too frequent relative to the timescales over which equilibrium is thought to emerge (Heaney 2000; Whittaker *et al*. 2008).

Diversity‐dependence, a negative feedback of diversity on diversification and immigration, has been proposed as a mechanism that facilitates the emergence of equilibrium species richness of clades or entire communities on islands and continents (Marshall *et al*. 1982; Rabosky & Glor 2010; Valente *et al*. 2014). However, whether the biotas of remote islands are sufficiently close to saturation for diversity feedbacks to have a discernible impact on community assembly is not known (Gillespie & Baldwin 2010; Rabosky & Glor 2010). Similarly, it remains an open question whether classic insular adaptive radiations [e.g. Darwin's finches (Grant & Grant 2008), African lake cichlids (Wagner *et al*. 2012) and Hawaiian Silverswords (Baldwin & Sanderson 1998)] represent exceptional evolutionary events decoupled from the processes governing the diversification of other lineages on islands or, instead, constitute unremarkable outcomes, given background rates of species gain and loss (Raup *et al*. 1973; Gillespie & Baldwin 2010; Etienne & Haegeman 2012).

Molecular phylogenies are a promising source of information regarding the temporal dimension of diversification and immigration on islands (Emerson 2002; Valente *et al*. 2014). Studies on insular environments have made use of phylogenetic trees to extract information on the accumulation of species through time (Ricklefs & Bermingham 2001; Rabosky & Glor 2010) and the contribution of *in situ* speciation (Kisel & Barraclough 2010; Papadopulos *et al*. 2011; Wagner *et al*. 2012) but the field of island biogeography lacks a phylogeny‐based inference approach that includes contributions from the three stochastic processes that determine diversity over evolutionary time scales – immigration, extinction and *in situ* speciation (Heaney 2000; Rosindell & Phillimore 2011). Here, we develop DAISIE (Dynamic Assembly of Islands through Speciation, Immigration and Extinction), a likelihood‐based phylogenetic method that unifies the island biogeography framework of MacArthur & Wilson (1963, 1967) with the phylogenetic birth‐death models popularised by Nee *et al*. (1992). DAISIE estimates diversity limits and per lineage rates of immigration, cladogenetic speciation (where one species on the island or archipelago splits into two endemic species), anagenetic speciation (where an island population diverges from its mainland source population through time) and extinction of insular biota. The method enables us to test whether the system has reached dynamic equilibrium as well as whether *in situ* radiations are exceptional given estimated background rates of biota assembly.

We apply DAISIE to the terrestrial avifauna of the remote Galápagos islands, a model system for evolutionary studies since Charles Darwin's visit in 1835. The Galápagos are renowned for their high avian endemicity but also for comprising species with diverse evolutionary and biogeographical backgrounds (Parent *et al*. 2008). The archipelago's avifauna includes the *in situ* radiations of 15 Darwin's finches [hereafter DF (Grant & Grant 2008; Farrington *et al*. 2014)] and four Galápagos mockingbirds (Lovette *et al*. 2012), as well as several endemic species that have no relatives on the archipelago, and taxa that have not differentiated substantially from their mainland ancestors (non‐endemic species). On the basis of the Galápagos remoteness (960 km from the South American continent) and young age [4–14 million years (My) (Werner *et al*. 1999; Geist *et al*. 2014)] it has been conjectured that diversity in the archipelago is below the equilibrium level (Heaney 2000; Parent & Crespi 2006), but this remains untested, and if rates of diversification and immigration have been high it is entirely plausible that equilibrium could have been achieved. Furthermore, although the adaptive radiation of DF is a textbook example of rapid evolution following colonisation of an ecologically vacant environment (Grant & Grant 2008; Farrington *et al*. 2014), it is possible that they diversified at the average background rates for the archipelago and belong to the high‐diversity tail of the distribution of outcomes from a shared macroevolutionary process (Raup *et al*. 1973; Rabosky *et al*. 2007) rather than being an exceptional radiation. Here, we estimate rates of assembly of the Galápagos terrestrial avifauna and show that total species diversity has not reached equilibrium. In addition, we show that Darwin's finches are decoupled from the background rates in the archipelago, and that they in fact appear to have reached a diversity steady state.

## Materials and Methods

### Galápagos land birds

**Figure 1:**
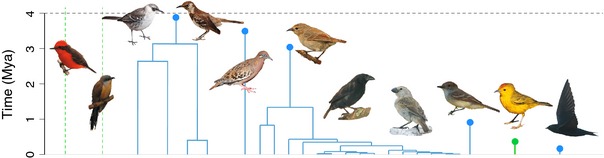
Colonisation and branching times of Galápagos terrestrial birds. Circles represent mean age of colonisation across all phylogenetic data sets. Blue – endemic lineages; green – non‐endemic lineages. Dashed lines are shown for the vermilion flycatcher and dark‐billed cuckoo as for these species only an upper bound to the age of colonisation is known. The dashed black line shows the approximate age of the oldest currently emerged island in the Galápagos archipelago. The photographs (by Ruben Heleno, Luis Valente and Steve Arlow) show representatives of each of the independent colonisation events. Lineage names are given in Table S1 in the same order as in the figure.

The Galápagos archipelago harbours 31 species of native resident land birds, comprising a diverse assemblage of avian families (Jiménez‐Uzcátegui *et al*. 2014). In order to analyse taxa with comparable ecological traits, we excluded birds of prey and rails (six species). Our data set thus includes 25 species corresponding to eight independent colonisations of the archipelago (Table S1). Three species are non‐endemic, found also in North, Central and South America. A total of 19 species belong to the two *in situ* land bird radiations that have taken place in the Galápagos, one giving rise to the DF and the other to the four species of Galápagos mockingbirds. We extracted colonisation and branching times from dated molecular phylogenies for all 25 species in order to reconstruct the pattern of species accumulation in the archipelago from its origin until the present (Fig. 1, Table S1).

Phylogenetic data sources and dating analyses are described in Supporting Information Methods and Tables S1–S7.

### Model

We modelled assembly of island biota as a stochastic process involving immigration (at per‐species rate γ) from a mainland with *M* species of unchanging identity, anagenesis (at per‐species rate λ^a^), *in situ* cladogenesis (at per‐species rate λ^c^) and island extinction (at per‐species rate μ).

We allow for diversity‐dependent diversification and immigration rates for each lineage, such that these rates decline linearly as the number of species on the island accumulates controlled by the parameter *K'*, which can be interpreted as the maximum number of species niches in the absence of extinction (Etienne *et al*. 2012). Mainland species can be treated as independent from one another, allowing separate treatment of the dynamics for each of the *M* mainland species. The first event, if any, that occurs is an immigration event, after which the immigrant can (1) diversify, either by anagenesis or cladogenesis (in either case the immigrant species status is changed, (2) it can become extinct or (3) it can immigrate again. If no endemic species have formed, then the later immigrant is assumed to replace the previous one, and the more recent colonisation event will be treated as the time of divergence from the mainland. This is crucial because in our inference from real data the time of colonisation for an anagenetic species or insular radiation is set at the divergence time from the mainland ancestor (see below).

### Likelihood computation

To estimate the rates of diversification and immigration and the diversity‐dependence parameter *K'*, we developed a method to compute the likelihood of the model given colonisation and branching times (obtained from dated phylogenies) of a biological group of interest from a focal region (from here on simply referred to as ‘island’). The data consist of a list of data entries, each of which refers to a different colonisation. We distinguish five types of data entries:

1. A mainland species that did not leave any extant island descendants. The mainland species may nevertheless have immigrated, and even diversified, but eventually the clade it established became extinct. The potential colonisation event is assumed to have occurred at any time after the island arose. The likelihood computation integrates over all possible colonisation times, and possible trajectories after colonisation.
2. A mainland species that is present on the island, but has no endemic sisters on the island, and the actual colonisation event is unknown. The colonisation event is then assumed to have occurred at any time after island birth (or divergence from a sister species if this is more recent), and the likelihood computation integrates out all possible trajectories.
3. Same as 2, but the actual colonisation event is known from the divergence time of the island population from the mainland population.
4. A mainland species that is not present on the island, but has left endemic descendants on the island, for which the time‐calibrated phylogeny is given. The colonisation event is set at the divergence time of the endemic clade from the closest mainland relative. Re‐immigration of the mainland species and subsequent extinction may have happened after the first anagenesis or cladogenesis event. The likelihood computation involves computing the probability of the phylogeny of the endemic clade.
5. A mainland species that co‐occurs on the island with its endemic sister clade, for which a phylogeny is provided. This means that the non‐endemic species re‐immigrated after one or more endemic species were formed. As in the previous case, the likelihood computation involves computing the probability of the phylogeny of the endemic clade.

The full likelihood is the product of the probabilities of observing these data entries under the model. These probabilities are computed, using a Hidden Markov approach (Etienne *et al*. 2012). Let us define $Q_{n}^{k}\left(t\right)$ as the probability that the process at time *t* is consistent with *k* lineages in the phylogeny at time *t*, and with *n* species that are not in the phylogeny assuming that the mainland species is absent. We define $Q_{n}^{M,k}\left(t\right)$ analogously but now assuming that the mainland species is (also) present (hence the superscript M). In the cases 1, 2 and 3 above where there is no tree, but simply the immigrant absent or present, we always have *k *=* *0 or *k *=* *1, respectively. In cases 4 and 5, *k* increases from 0 to 1 at the colonisation event, and increases by 1 at each branching point in the tree. When *k *=* *0 or *k* > 1 the probabilities $Q_{n}^{k}\left(t\right)$ and $Q_{n}^{M,k}\left(t\right)$ can be obtained by integrating the following set of differential equations (master equations):

(1) $$\begin{array}{cc} \frac{dQ_{n}^{k}}{dt} & =\mu Q_{n}^{M,k}+\lambda^{a}Q_{n-1}^{M,k}+\lambda_{n+k-1}^{c}Q_{n-2}^{M,k}\\ & +\lambda_{n+k-1}^{c}\left(n+2k-1\right)Q_{n-1}^{k}+\mu \left(n+1\right)Q_{n+1}^{k}\\ & \;-\left(\mu +\lambda_{n+k}^{c}\right)\left(n+k\right)Q_{n}^{k}-\gamma_{n+k}Q_{n}^{k}\\ \frac{dQ_{n}^{M,k}}{dt} & =\gamma_{n+k}Q_{n}^{k}+\lambda_{n+k}^{c}\left(n+2k-1\right)Q_{n-1}^{M,k}\\ & \;+\mu \left(n+1\right)Q_{n+1}^{M,k}-\left(\mu +{\lambda^{c}}_{n+k+1}\right)\left(n+k\right)Q_{n}^{M,k}\\ & \;-\left(\mu +\lambda^{a}+{\lambda^{c}}_{n+k+1}\right)Q_{n}^{M,k} \end{array},$$

subject to initial conditions $Q_{0}^{0}\left(0\right)=1,Q_{0}^{M,0}\left(0\right)=0\;\text{and}Q_{n}^{k}\left(0\right)=Q_{0}^{M,k}\left(0\right)=0$ for all other combinations of *k* and *n*. We emphasise that all the parameters of the model may depend on the number of species in the colonising lineage, thus incorporating diversity‐dependence (although we only considered such a dependence for the rates of cladogenesis and immigration); we chose not to make this dependence explicit in our equations for notational convenience. At the branching points the probabilities $Q_{n}^{k}\left(t\right)$ and $Q_{n}^{M,k}\left(t\right)$ are updated to take into account the speciation event:

(2) $$\begin{array}{cc} Q_{n}^{k+1}= & \lambda_{n+k}^{c}Q_{n}^{k}\\ Q_{n}^{M,k+1}= & \lambda_{n+k+1}^{c}Q_{n}^{M,k} \end{array}$$

As stated above, this applies only to *k *= 0 and *k* > 1. The case *k *=* *1 is special because this case arises right after the colonisation event that defines the tree (or single surviving lineage). To be consistent with the phylogeny there cannot be any re‐immigration before the lineage has speciated because re‐immigration before speciation would reset the divergence time. So let us define $Q_{M,n}\left(t\right)$ as the probability that the mainland species whose lineage will survive to the present is present on the island at time *t* with *n* other species that will not survive, or are not in the phylogeny. The probabilities $Q_{n}^{1}\left(t\right)$, $Q_{n}^{M,1}\left(t\right)$ and $Q_{M,n}\left(t\right)$ can be obtained by solving the following system of differential equations:

(3) $$\begin{array}{cc} \frac{dQ_{n}^{1}}{dt}= & \lambda^{a}Q_{M,n}+2\lambda_{n}^{c}Q_{M,n-1}+\mu Q_{n}^{M,1}+\lambda^{a}Q_{n-1}^{M,1}+\lambda_{n}^{c}Q_{n-2}^{M,1}\\ & +\lambda_{n}^{c}\left(n+1\right)Q_{n-1}^{1}+\mu \left(n+1\right)Q_{n+1}^{1}\\ & -\left(\mu +\lambda_{n+1}^{c}\right)\left(n+1\right)Q_{n}^{1}-\gamma_{n+1}Q_{n}^{1}\\ \frac{dQ_{n}^{M,1}}{dt}= & \gamma_{n+1}Q_{n}^{1}+\lambda_{n+1}^{c}\left(n+1\right)Q_{n-1}^{M,1}+\mu \left(n+1\right)Q_{n+1}^{M,1}\\ & -\left(\mu +\lambda_{n+2}^{c}\right)\left(n+1\right)Q_{n}^{M,1}-\left(\mu +\lambda^{a}+\lambda_{n+2}^{c}\right)Q_{n}^{M,1}\\ \frac{dQ_{M,n}}{dt}= & \mu \left(n+1\right)Q_{M,n+1}+\lambda_{n}^{c}\left(n-1\right)Q_{M,n-1}\\ & -\left[\left(\mu +\lambda_{n+1}^{c}\right)\left(n+1\right)-\left(\lambda^{a}+\gamma_{n+1}\right)\right]Q_{M,n} \end{array},$$

with the initial condition at the colonisation time *t* = *t*_c_ being $Q_{n}^{1}\left(t_{c}\right)=0$ *,* $Q_{n}^{M,1}\left(t_{c}\right)=0$ *,* $Q_{M,n}\left(t_{c}\right)=\gamma_{n}Q_{n}^{0}\left(t_{c}\right)$ where $Q_{n}^{0}\left(t_{c}\right)$ is computed from Eq (1), so all probabilities $Q_{n}^{0}\left(t\right)$ are transferred to *Q*_M,*n*_(*t*). At the node where *k* becomes 2, we have

(4) $$\begin{array}{cc} & Q_{n}^{2}=\lambda_{n+1}^{c}\left(Q_{n}^{1}+Q_{M,n}\right)\\ & \;Q_{n}^{M,2}=\lambda_{n+2}^{c}Q_{n}^{M,1} \end{array},$$

so all probabilities *Q*_M,*n*_(*t*) are transferred to $Q_{n}^{2}\left(t\right)$.

The likelihood is eventually given by $Q_{n}^{k}\left(t_{p}\right)$ or $Q_{n}^{M,k}\left(t_{p}\right)$ *,* depending on whether the mainland species is absent or present, where *t*_p_ is the present time and *k* equals the number of species in the phylogeny (where *k *=* *0 constitutes an empty phylogeny, and *k *=* *1 a single stem) and *n* is the number of descendants of the colonist for which we do not have phylogenetic data.

As a technical detail, we remark that events where the mainland species reimmigrates and establishes a new clade are taken into account in the computation of the probabilities $Q_{n}^{M,k}\left(t\right)$ by adding the species in the new clade to the *n* species that are not in the phylogeny. We do not, therefore, compute probabilities for the branching pattern of the new clade as we consider these events to be rare and they did not occur in our data, but we do account for the contribution of these species to diversity‐dependence.

We implemented the model, likelihood estimation framework and simulation code in a new R package called DAISIE (available from the R website, http://cran.r-project.org/web/packages/DAISIE/).

### Model fitting and selection

Model parameters for the Galápagos phylogenetic data set were estimated via maximum likelihood in the DAISIE package. We compared equal‐rates DAISIE models that assume that all lineages share the same dynamics to differential‐rate models that distinguish DF‐type species (defined as those mainland finch species that have the potential to produce a DF‐type radiation) from the remaining bird taxa in the archipelago (non‐DF‐type species), thus allowing the DF radiation to differ in one or more parameters that govern the macroevolutionary process (Table S8). While it is possible that not all non‐DF‐type species share the same parameters, we chose to compare DF and non‐DF‐type species because we were interested in the specific question of whether DFs are an exceptional radiation.

In order to estimate overall rates of species accumulation for the Galápagos (treating the archipelago as an island) we fitted two equal‐rates models that assume that a single macroevolutionary process applies to all lineages (M1 and M1′). The models we fitted are described in detail in Table S8. All differential‐rate models tested have additional parameters compared to the equal‐rates models, corresponding to the parameters that are allowed to vary between DF‐type and non‐DF‐type species. For the differential‐rate models, the proportion of DF‐type species (*P*_DF‐type_) in the mainland pool was assumed to be 0.163, equivalent to the proportion of extant finch‐like species in the South American avifauna. To compute *P*_DF‐type_, we referred to a complete checklist of South American bird species (Birdlife International) and divided the total number of potential DF‐type (Thraupidae, Emberizidae and Fringillidae) by the total number of land birds representing broadly comparable guilds to the Galápagos avifauna. We justify this broad definition of potential finch ancestors based on the observation that finches have radiated on two other archipelagos (Price 2011). However, we also assessed the robustness of our results with respect to changing *P*_DF‐type_ (see Sensitivity analysis section).

We fitted all models to a ‘consensus’ data set representing the mean colonisation and branching times across all phylogenetic trees. For each model, we ran optimisations with 100 different initial starting conditions to minimise the chance of being trapped in local suboptima. We repeated analyses assuming *M *=* *500 and 1000 species in the mainland species pool, but report only the results for *M *=* *1000 because parameter estimates varied little with pool size, except for γ, which, as expected, was smaller with higher *M*. We compared models using Akaike information criterion (AIC) and Bayesian information criterion (BIC) weights (*n *= *M*), and found that model M8 was strongly preferred using both criteria. We only report results using BIC, as this criterion was found to have very low type I error rates with simulated data.

### Sensitivity analyses

In order to account for phylogenetic uncertainty, we repeated analyses for the three best models (M1, M5 and M8) across 100 randomly sampled phylogenetic data sets thus incorporating variation in node ages. The oldest currently emerged island in the Galápagos is *c*. 4 My old, but seamounts representing drowned islands anciently formed above the Galápagos hotspot suggest the archipelago could be up to 14.5 My old (Werner *et al*. 1999; Geist *et al*. 2014). To account for uncertainty in geological age, we refitted the three best models assuming a range of archipelago ages between four and 15 My.

The fraction of DF‐types on the mainland (*P*_DF‐type_) of 0.163 used in the main analysis may be an overestimate, because DFs are derived from a Caribbean clade (Burns *et al*. 2002) that is not necessarily typical of South American finch‐like species. We therefore refitted the three best models assuming *P*_DF‐type_ values varying between 0.001 and 0.28 (the maximum value is the *P*_DF‐type_ in South America if only lineages present on the Galápagos are taken into account), in order to examine the effect of *P*_DF‐type_ on our main conclusions.

There are currently no molecular data available for one of the species in our data set – *Progne modesta*. To evaluate the effect of the colonisation time of *P. modesta* on our parameter estimates, we refitted the best model with varying colonisation times of *P. modesta* between 0.01 and 4 Mya.

### Simulations

Using a standard algorithm that allows stochastic simulations in continuous time (Gillespie 1976), we simulated 5000 islands under the ML parameter estimates from the best model (M8), covering the entire life span of the archipelago from birth (4 Ma) to the present. To assess whether the model faithfully reproduces the observed Galápagos phylogenetic and diversity data, we plotted the distribution of various summary statistics. The actual age of colonisation of two non‐endemic species (*Coccyzus melacoryphus* and *Pyrocephalus rubinus*) is unknown, which we accounted for in our model fitting by integrating over all possible ages. To check whether this results in reasonable colonisation times, we estimated median times of colonisation of non‐endemic species predicted under the model by simulating lineages with the ML parameters of non‐DF‐type species and recording the time of colonisation of lineages that were present on the island but had not yet speciated at the end of the simulations.

### Precision estimates

To assess the performance of our likelihood inference method, we used a parametric bootstrap approach to measure the ability of the model to estimate the correct parameters from data sets simulated with known values. We performed simulations of the M8 model using the ML parameter estimates from the data as input. We then assessed bias and precision by estimating the ML parameters from each of the simulated data sets and comparing them with the simulated values.

## Results

### Model selection

**Figure 2:**
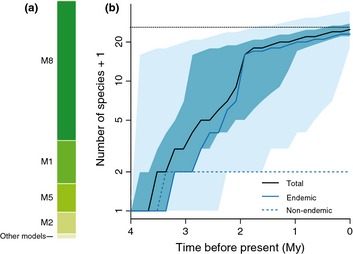
Diversity‐through‐time of the Galápagos terrestrial avifauna (a) Bayesian information criterion weights obtained by fitting different models to the consensus Galápagos phylogenetic data set reveal strong support for the M8 model. (b) Total number of species through time obtained in 5000 stochastic simulations of the M8 model, conditioning on there being a single DF colonisation. Lines show medians, and light and dark blue areas show respectively the 2.5th and 75th percentiles across the 5000 simulated data sets. Dotted black line shows the number of observed species in the Galápagos data. Model names defined in Table S8.

**Figure 3:**
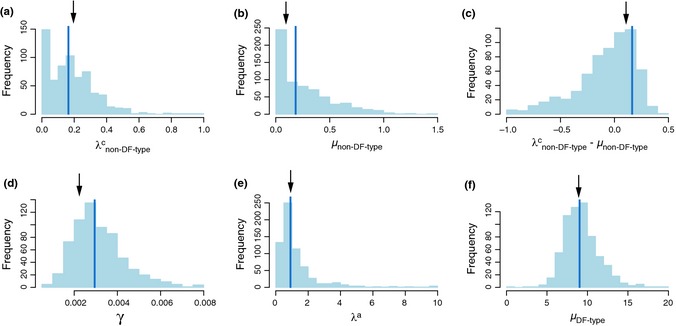
Precision estimates of the M8 model. Results of the parametric bootstrap analysis obtained by fitting the M8 model to 2000 data sets simulated with the ML parameters of the M8 model for the Galápagos phylogenetic data set. Plots are frequency histograms of rates of (a) cladogenesis of non‐DF‐type species (λ^c^_non‐DF‐type_), (b) extinction of non‐DF‐type species (μ_non‐DF‐type_), (c) diversification of non‐DF‐type species (λ^c^_non‐DF‐type_ − μ_non‐DF‐type_), (d) immigration (γ), (e) anagenesis (λ^a^) and (f) extinction of DF‐type species (μ _DF‐type_). Blue lines show the median estimated value across all simulations and black arrows the (true) value used in simulations.

The dated phylogenetic trees of Galápagos land birds indicate that the majority of colonisations and all speciation events are younger than the age of the oldest currently emerged island (Fig. 1). The most strongly supported model for the Galápagos data set (M8 in Table S8; results in Fig. 2a and Table S9) allows for a decoupling of the dynamics experienced by DF‐type from the background rate, such that DF‐types are subject to diversity‐dependence and a higher rate of cladogenesis and extinction than non‐DF‐type species. Non‐DF‐type species accumulate species at slow rates and experience no diversity‐dependent feedbacks (Table S9). Under the M8 model the rate of immigration to the archipelago is estimated to be low and anagenesis plays an important role in generating endemism (Table S9). DF species show an explosive burst of cladogenesis, rapid extinction and a diversity limit of 15 species. Under these parameters, the rate of cladogenesis of the DF radiation is nearly instantaneous, the carrying capacity is reached very rapidly and any extinction event is quickly followed by a cladogenesis event, thus resembling a coalescent‐like process (Hey 1992). Parametric bootstrap revealed that the method performs well in recovering the correct parameter values with very little bias (Fig. 3, Fig. S1 and Table S10).

### Simulations

**Figure 4:**
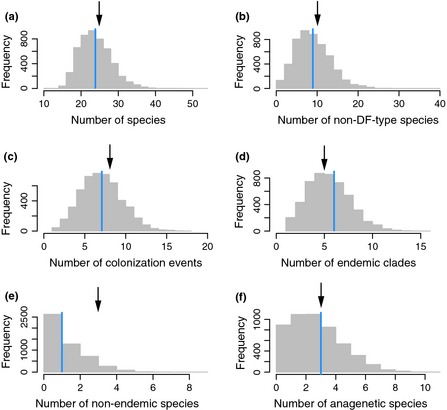
Goodness of fit of the M8 model. Histograms show the number of (a) total species, (b) non‐DF‐type species, (c) colonisation events, (d) endemic clades, (e) non‐endemic clades and (f) anagenetic species in the 5000 phylogenetic data sets simulated with the ML parameters of the M8 model. Blue vertical lines indicate median values across simulated data sets and black arrows indicate the observed values in the Galápagos data.

Diversity‐through‐time plots produced by simulating species accumulation from the age of the oldest currently emerged island (4 My) until the present using M8 parameter estimates show that Galápagos land birds have not attained an equilibrium diversity, as total species richness does not exhibit asymptotic behaviour (Fig. 2b). The simulations also revealed that the model provides a very good fit to the data (Fig. 4) and that the median time of colonisation of non‐DF‐type non‐endemic species is 0.58 (0.04–2.4) Mya (Fig. S2)

### Sensitivity analyses

A sensitivity analysis revealed that our conclusion that total species diversity is not at equilibrium is robust to variation in node ages (Table S11) and assumed Galápagos geological age (Table S12 and Fig. S3a). Varying *P*_DF‐type_ values (Table S13 and Fig. S3b) or the assumed age of colonisation of *P. modesta* (Table S14) led to changes in biogeographical rates but did not alter the conclusion that DF‐types appear to be at equilibrium while total avian diversity is not.

## Discussion

According to the most strongly supported DAISIE model, the avifauna of the Galápagos islands does not tend towards a diversity steady state where the contributions of immigration and speciation are balanced by extinction, contrary to the expectations of classic ecological equilibrium theory (MacArthur & Wilson 1963, 1967). Departure from equilibrium expectations in the Galápagos terrestrial avifauna (Fig. 2b) occurs because non‐DF‐type species exhibit a positive diversification rate and no detectable signal of diversity‐dependent controls, and thus may be fundamentally non‐equilibrial (Quental & Marshall 2013; Warren *et al*. 2015). Our analyses suggest that the archipelago has been continuously accumulating species at slow rates, through both new arrivals and *in situ* speciation, and total diversity is far from achieving an asymptotic phase.

In contrast, a subset of the avifauna – DF‐type species – appears to have reached a diversity limit and can be considered to be in an equilibrium steady state, according to the best model. Our analyses thus suggest that, unless new key innovations arise (Etienne & Haegeman 2012), the DF radiation may have reached an end point in terms of numbers of species, even though evolutionary turnover of finch taxa is estimated to be remarkably high. The diversity limit that we propose operates on DF requires diversity‐dependence to occur on an archipelago‐wide scale where species ranges may not overlap. Negative diversity feedbacks may operate in both sympatric and highly fragmented allopatric settings (Rabosky & Lovette 2008; Pigot *et al*. 2010; Moen & Morlon 2014; Price *et al*. 2014), and, indeed, numerous studies have found evidence for diversification slowdowns and diversity‐dependence in clades composed of allopatric forms (Rundell & Price 2009). In the case of DF, we suggest that the carrying capacity emerges as a function of limits to both alpha and beta diversity – thus, if a form distributed over several islands splits into two species with smaller ranges, each of these forms will then have a higher probability of extinction (Pigot *et al*. 2010). Given that DF constitute the most extensively studied avian radiation, and that sampling has been very comprehensive throughout the entire geographical range (Grant & Grant 2008; Farrington *et al*. 2014; Lamichhaney *et al*. 2015), we believe it is unlikely that the detected diversity limit is the result of a failure to recognise incipient species (Etienne & Rosindell 2012).

The exceptional nature of the DF radiation is illustrated not only by their differential rates of cladogenesis, extinction and the presence of diversity limits but also by their coalescent‐like diversification (Hey 1992), a process that typically is modelled at the population rather than the species level, but see Barraclough (2010) and Humphreys & Barraclough (2014). This supports the hypothesis suggested by long‐term ecological and evolutionary studies that traditional speciation models may not apply to the DF radiation (Grant & Grant 2008). Moreover, our results suggest that DF‐type‐species possess characteristics that triggered a rapid evolutionary divergence (Grant & Grant 2008) in an environment that has not been conducive to such rapid diversification of other colonising lineages. These insights agree with the observation that finch‐like species have a tendency to radiate on islands, such as Hawaii or Tristan da Cunha archipelagoes (Price 2011). Our study thus challenges the view that radiations are an inevitable feature of ecologically vacant remote archipelagoes (MacArthur & Wilson 1963; Heaney 2000), and suggests that isolation and ecological opportunity must combine with the colonisation of species that possess particular traits in order for radiations to arise (Gillespie & Baldwin 2010; Etienne & Haegeman 2012; Wagner *et al*. 2012).

The fact that total avian diversity of the Galápagos archipelago has not achieved a steady state after at least 4 My of existence questions the adequacy of an equilibrium perspective for understanding biodiversity patterns on remote oceanic islands (MacArthur & Wilson 1963; Heaney 2000; Gillespie & Baldwin 2010; Triantis *et al*. 2015). The absence of a signature of diversity‐feedbacks and ecological limits in non‐DF‐type species in the Galápagos contrasts with recent studies on less isolated biodiverse landmasses that have detected slowdowns within most clades, consistent with ecological controls to diversity (Rabosky & Glor 2010; Scantlebury 2013). Our finding that non‐DF‐type species in the Galápagos, including the small radiation of mockingbirds, are fundamentally non‐equilibrial, may be a consequence of the relatively low avian diversity and species packing in the Galápagos when compared with other tropical archipelagos. This may imply that diversity controls are relaxed in isolated and ephemeral environments, or that rather than diversity‐dependence operating within each colonising lineage, as most macroevolutionary studies assume, the entire biota may subject to the same diversity‐dependent regulation in the same arena. Indeed, whilst Darwin's hypothesis that competition is strongest between close relatives has been experimentally confirmed (Violle *et al*. 2011), it may not be ubiquitous (Narwani *et al*. 2013).

There has recently been increased recognition of the role of island ontogeny and sea‐level fluctuations in shaping diversification and colonisation rates on insular systems through the effects of these phenomena on island area and connectivity (Whittaker *et al*. 2008; Ali & Aitchison 2014; Gillespie & Roderick 2014; Valente *et al*. 2014). In our study, we have treated the Galápagos as a single island and have assumed that total area of the archipelago has remained relatively constant throughout the last 4 My. We have based this assumption on the high geological dynamism of the Galápagos volcanic hotspot, illustrated by rapid and continuous formation and submergence of multiple islands and cyclical fragmentation and fusion of landmasses (Ali & Aitchison 2014; Geist *et al*. 2014). Geological evidence suggests that at least seven major islands have existed in the archipelago since the formation of the oldest currently emerged island, albeit in different configurations to the contemporary geographical arrangement (Geist *et al*. 2014). Because of this continuous emergence and submergence, the assumption of overall area‐independent diversification rate may be a fair approximation. Note that the stochasticity of our model already allows for such ‘noise’ to some extent. The assumption of constant rates in the face of observations of apparent fluctuations has been extensively discussed by Raup *et al*. (1973). Explicit incorporation of the area trajectories of multiple islands that differ in age and whose ontogeny is known (e.g. using the approach of Valente *et al*. 2014), may in the future allow testing of the effect of area on equilibrium dynamics in the Galápagos. However, this would require several additional parameters, the estimation of which may only be feasible in the context of a multiple rather than single island model context. We expect that certain bird taxa (such as many non‐DF‐type taxa in our data set that occur on most islands of the Galápagos and have not diversified) may require more isolation than the archipelago can provide in order to produce new incipient species *in situ*, even in periods of peak geographical complexity.

Investigations of the pattern of species assembly on island‐like systems using phylogenies have typically focused on lineage‐specific dynamics rather than encompassing entire insular communities, and most studies tend to overlook colonisation events that have not produced endemic species or *in situ* radiations (Warren *et al*. 2015). Hence, phylogenetic data sets with across‐lineage sampling of members of a higher taxon, ecological guild or trophic level comprising independent colonising lineages in the same insular system are still rare, especially for remote settings. At present, the Galápagos avian data set is the only one that meets these criteria. While it has only a moderate number of avian species compared to other more species‐rich communities, we have made a thorough examination of the ability of our framework to correctly estimate parameters and distinguish between models, which convincingly showed that our data set is large enough to produce robust conclusions. We hope that the availability of DAISIE will encourage island biologists to collect phylogenetic data sets that encompass whole communities and include estimates of stem ages of independent island lineages, thus enabling tests of equilibrium dynamics over evolutionary time scales on a variety of insular systems.

In conclusion, our key finding that both equilibrium and non‐equilibrium dynamics can simultaneously operate within the same community within a single geographical region, calls for a re‐examination of equilibrium theory in order to incorporate across‐lineage heterogeneity in the processes that govern species accumulation. We provide a framework (DAISIE) for testing for equilibrium on isolated communities incorporating immigration, speciation and extinction, thereby offering new opportunities for islands to remain natural laboratories furthering our understanding of insular community assembly.

## Authorship

All authors contributed to conceptual framework, model development, interpretation of results and manuscript writing. ABP and LMV initiated the research. RSE developed the likelihood approach. LMV compiled the phylogenetic data and wrote the first draft. LMV and RSE conducted the analyses.
