# Supplementary material for: Equilibrium and non‐equilibrium dynamics simultaneously operate in the Galápagos islands
Source: Ecol Lett. 2015 Jun 23;18(8):844–52. doi: 10.1111/ele.12461 (PMC4745040; doi:10.1111/ele.12461)
Supplement: Supplementary file 2 [file ELE-18-844-s002.docx]

**Online supporting information**

*Ecology Letters* Article:

**Equilibrium and non-equilibrium dynamics simultaneously operate in the Galápagos islands**

Luis M. Valente, Albert B. Phillimore, Rampal S. Etienne

**Contains:**

Supplementary Methods

Supplementary Figures S1-S3

Supplementary Tables S1-S14

Supporting Information References

**Supplementary methods**

**Galápagos phylogenetic/phylogeographic studies**

We obtained phylogenetic data based on two sources. Firstly, we re-analysed molecular phylogenetic and phylogeographic data from published studies that have specifically targeted Galápagos taxa (Table S1). Secondly, for the three taxa for which such studies were unavailable, we used a time-calibrated global phylogeny of all avian taxa (Jetz *et al.* 2012).

We searched the literature for studies that have used molecular data to reconstruct the biogeographical and evolutionary history of Galápagos land bird taxa (populations in the case of non-endemic Galápagos species, or species in the case of endemic species). We sought to identify the closest non-Galápagos relatives of Galápagos taxa and/or the timings of colonisation and speciation within the archipelago. Informative studies were available for five independent colonisation events, comprising 22 Galápagos land bird taxa. We obtained DNA sequences for each of these species plus appropriate outgroup taxa and built time-calibrated phylogenetic trees for each independent colonisation event (including the phylogeny of the entire radiation in the case of the DF and the Galápagos mockingbirds). GenBank accession numbers are provided in Tables S2-S6, while locality and voucher information are reported in the GenBank records. Ultrametric trees were produced in BEAST (Drummond & Rambaut 2007) v1.8.0 using the best model of molecular evolution as selected using the AIC in jModeltest (Table S7). In the absence of pre-Holocene Galápagos land bird fossils (Steadman *et al.* 1991), we performed divergence dating using rates of molecular evolution for avian mitochondrial sequences, which have been shown to evolve in a clock-like fashion at an average rate of 2.1% per million years (Weir & Schluter 2008). We applied a Bayesian relaxed uncorrelated clock model, and for each analysis we employed the average molecular rate of evolution of mitochondrial sequences estimated in the literature for the respective clade (Table S7). For each analysis, we ran four independent chains of 30 million generations, setting a birth-death tree prior. Convergence of chains and appropriate burn-ins were assessed with Tracer and maximum clade credibility trees with mean node heights were produced in Tree Annotator.

For three of the eight colonisation events (*Coccyzus melacoryphus*, *Pyrocephalus rubinus* and *Progne modesta*) there are no published in-depth molecular studies that would allow us to confidently identify the closest extant relative of the Galápagos taxa. Therefore for these cases we used a global time-calibrated phylogenetic analysis of all bird species (Jetz *et al.* 2012) as a source of data on colonisation times of the Galápagos archipelago. We extracted posterior distributions of trees with subsets of taxa from the global phylogeny of birds using a tool available in [www.birdtree.org](http://www.birdtree.org) (this was repeated for “Hackett” and “Ericson” dating constraints but we report only the results for “Hackett” trees, as both analyses were congruent). The taxon subsets include all members of each of the bird families represented in the three colonisation events (Cuculidae, Hirundinidae and Tyrannidae). We extracted 5,000 posterior trees for each of these three bird families, covering a total of 648 species.

**Darwin’s finches**

DF gene trees are often incongruent and many species show poly/paraphyletic patterns, such that dating branching events within the finch radiation is notoriously challenging (Grant & Grant 2008; Farrington *et al.* 2014). Farrington and colleagues (Farrington *et al.* 2014) produced the most comprehensive DF species tree to date, which includes all recognized species of DF and is based on mitochondrial markers in combination with 14 nuclear introns. The authors produced ultrametric DF species trees using *BEAST, but did not estimate absolute dates. We added new outgroup taxa (Genbank accession numbers are reported in Table S6) to their mitochondrial dataset and dated it in BEAST using the same procedure described above for other Galápagos taxa. We then rescaled the posterior distribution of ultrametric species trees by applying the posterior distribution of the stem age of DF from the mitochondrial dating analysis to the stem age of the species trees based on both mitochondrial and nuclear data, preserving the order of stem ages.

**Colonisation and speciation times**

We assumed that the time of divergence of a Galápagos lineage (population, species or clade) from its closest related non-Galápagos lineage corresponds to the time of colonisation of the archipelago. For two non-endemic species (*Coccyzus melacoryphus* and *Pyrocephalus rubinus*) DNA sequences were only available from non-Galápagos populations and for these cases only a maximum age of colonisation is known. We thus used the divergence time of the entire species from their sister lineages as an upper bound for the age of the colonisation event (Table S1). This age is not used as an approximation – the DAISIE inference method integrates over the possible colonisation times between the present and the upper bound.

**
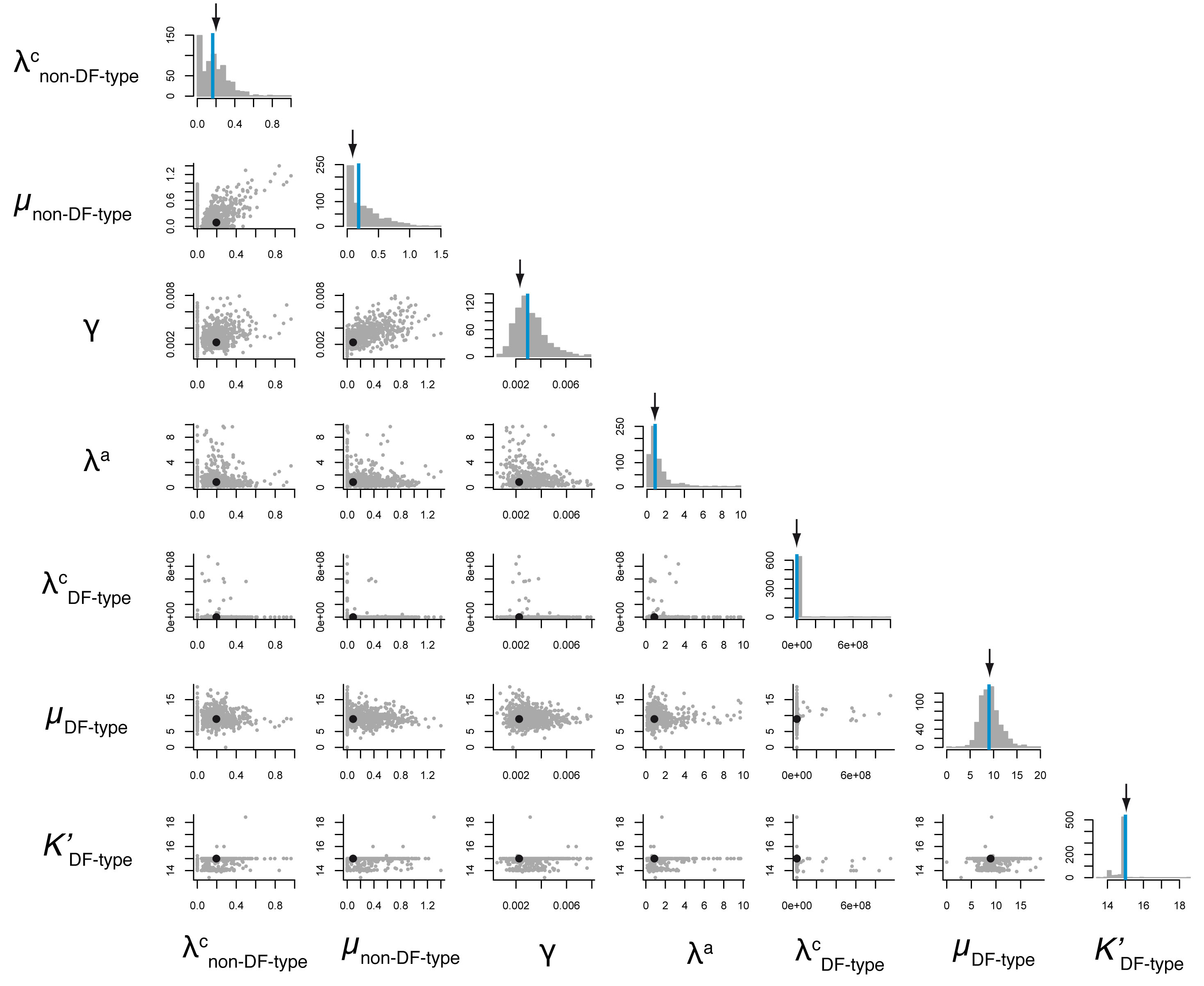
**

**Fig. S1 - Precision estimates of all parameters of the M8 model.** Results of the parametric bootstrap analysis obtained by fitting the M8 model to 2,000 datasets simulated with the ML parameters of the M8 model for the Galápagos phylogenetic dataset. Plots on the diagonal are frequency histograms of the estimated parameters. The black line shows the median estimated value across all simulations and the blue line the simulated value. Scatterplots show the relationship between the parameter shown at the start of the row to the parameter below the column for each simulated dataset. The black dot is the (true) value used in simulations. λ^c^ - per lineage rate of cladogenesis; μ - per lineage rate of extinction; γ - per lineage rate of immigration; λ^a^ - per lineage rate of anagenesis, *K*’ – carrying capacity.

**Fig. S2 – Colonisation times of non-endemic species without close relatives on the island, as predicted under the M8 model.** Results are from two million replicates of simulations of lineages with the ML parameters of non DF-type species under the M8 model for four million years.

**
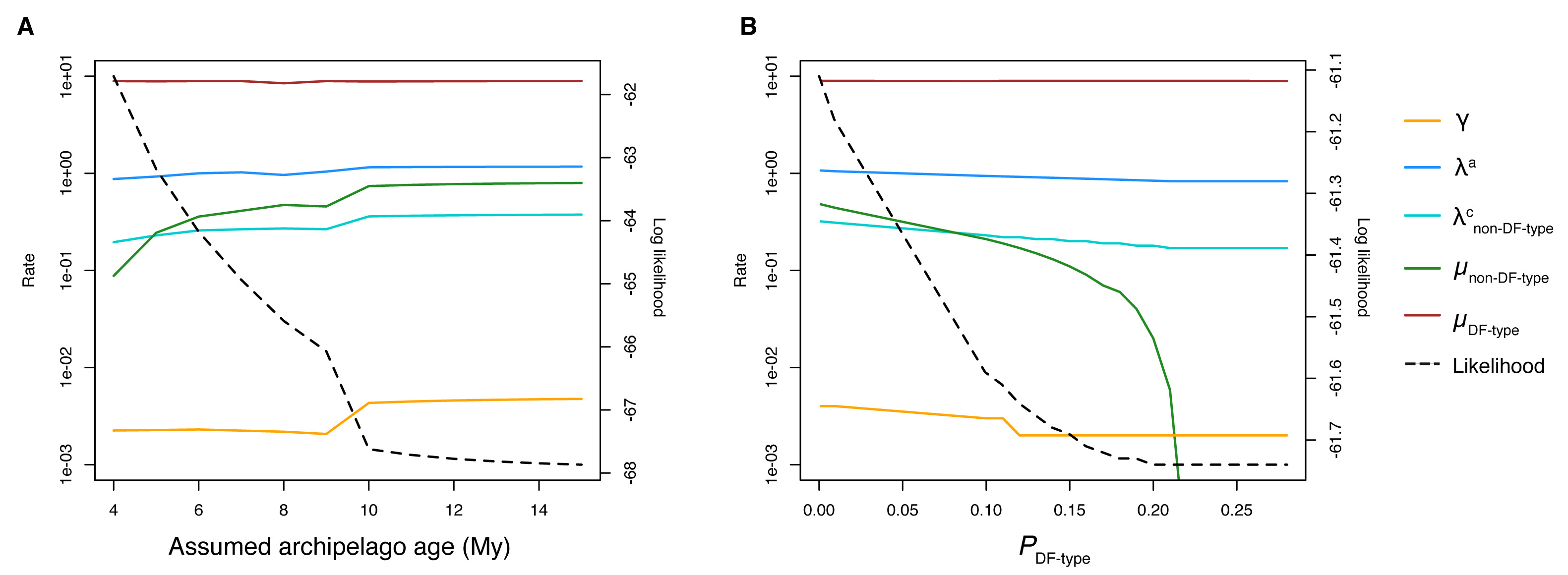
**

**Fig. S3 – Sensitivity analysis of the M8 model.** Effect of varying **(A)** assumed archipelago age, and **(B)** the proportion of DF-type species in the mainland pool (*P*_DF-type_), on maximum likelihood values and parameter estimates of the M8 model. γ - per lineage rate of immigration; λ^a^ - per lineage rate of anagenesis; λ^c^_non-DF-type_ - per lineage rate of cladogenesis of non-DF-type species; μ_non-DF-type_ - per lineage rate of extinction of non-DF-type species; μ_DF-type_ - per lineage rate of extinction of DF-type species.

**Table S1 - Colonisation time estimates for Galápagos native terrestrial bird lineages**. Ages shown are mean estimates and 95% highest posterior density across posterior distribution of trees from the BEAST output, except when noted.

| Lineage name | Galápagos species | Type | Stem age (Mya) | Data sources |
| --- | --- | --- | --- | --- |
| Vermilion flycatcher | *Pyrocephalus rubinus* | Non-endemic | 10.28 (4.53-12.91)*† | (Jetz *et al.* 2012) |
| Dark-billed cuckoo | *Coccyzus melacoryphus* | Non-endemic | 7.456 (3.97-11.65)*† | (Jetz *et al.* 2012) |
| Galápagos mockingbirds | Radiation (4 species) | Cladogenetic | 3.958 (3.35-4.55) | (Lovette *et al.* 2012) |
| Galápagos dove | *Zenaida galapagoensis* | Anagenetic | 3.51 (2.57-4.65) | (Johnson & Clayton 2000) |
| Darwin’s finches | Radiation (15 species) | Cladogenetic | 3.028 (2.22-3.85) | (Farrington *et al.* 2014) |
| Galápagos flycatcher | *Myiarchus magnirostris* | Anagenetic | 0.855 (0.58-1.13) | (Sari & Parker 2012) |
| Galápagos warbler | *Dendroica petechia aureola* | Non-endemic | 0.34 (0.187-0.50) | (Chaves *et al.* 2012) |
| Galápagos martin | *Progne modesta* | Anagenetic | 0.086 (0.002-0.32)* | (Jetz *et al.* 2012) |

Mya – millions of years ago.

* These estimates were obtained from the global avian time-calibrated phylogeny of Jetz *et al.* (2012) and we thus report the mean, 2.5^th^ and 97.5^th^ percentiles from 5,000 trees with the Hackett backbone.

† Maximum age of colonisation. DNA sequences from Galápagos individuals were not available and we thus use the divergence time of non-Galápagos individuals from the sister species as an upper bound for the time of colonisation. This age is not used as an approximation – the DAISIE inference method uses this maximum age to integrate over the possible colonisation times.

**Table S2**  **-** Genbank accession numbers used in Galápagos dove phylogenetic analyses.

| **Genus** | **Species** | **Cytochrome B** | **ND2** |
| --- | --- | --- | --- |
| *Zenaida* | *asiatica* | AF251534 | AF251543 |
| *Zenaida* | *auriculata* | AF483340 | AF251538 |
| *Zenaida* | *aurita* | AF182704 | AF251541 |
| *Zenaida* | *galapagoensis*_1 | AF182701 | AF251539 |
| *Zenaida* | *galapagoensis*_2 | AF251531 | AF251540 |
| *Zenaida* | *graysoni* | AF182702 | AF251537 |
| *Zenaida* | *macroura* | AF251530 | AF251535 |
| *Zenaida* | *meloda* | AF182699 | AF251545 |

**Table S3**  – Genbank accession numbers used in Galápagos flycatcher phylogenetic analyses.

| **Genus** | **Species** |  | **Cytochrome B** | **ND2** |
| --- | --- | --- | --- | --- |
| *Myiarchus* | *cinerascens* |  | JQ004298 | JQ004349 |
| *Myiarchus* | *crinitus* |  | JQ004299 | JQ004350 |
| *Myiarchus* | *magnirostris*_1 |  | JQ004300 | JQ004351 |
| *Myiarchus* | *magnirostris*_2 |  | JQ004301 | JQ004352 |
| *Myiarchus* | *magnirostris*_3 |  | JQ004302 | JQ004353 |
| *Myiarchus* | *magnirostris*_4 |  | JQ004303 | JQ004354 |
| *Myiarchus* | *magnirostris*_5 |  | JQ004304 | JQ004355 |
| *Myiarchus* | *sagrae* |  | JQ004313 | JQ004368 |
| *Myiarchus* | *tyrannulus*_1 |  | JQ004317 | JQ004372 |
| *Myiarchus* | *tyrannulus*_2 |  | JQ004318 | JQ004373 |
| *Myiarchus* | *tyrannulus*_3 |  | JQ004328 | JQ004383 |
| *Myiarchus* | *tyrannulus*_4 |  | JQ004333 | JQ004386 |
| *Myiarchus* | *yucatanensis* |  | JQ004341 | JQ004392 |
|  |  |  |  |  |

**Table S4**  – Genbank accession numbers used in Galápagos mockingbirds phylogenetic analyses.

| **Genus** | **Species** | **ATP** | **CO1** | **ND2** |
| --- | --- | --- | --- | --- |
| *Melanoptila* | *glabrirostris* | EF486781 | EF484221 | EF468197 |
| *Mimus* | *gilvus* | EF486780 | EF484220 | EF468196 |
| *Mimus* | *gundlachii* | EF486782 | EF484222 | EF468198 |
| *Mimus* | *longicaudatus* | EF486784 | EF484224 | EF468200 |
| *Mimus* | *macdonaldi* | DQ087196 | DQ083930 | AY311555 |
| *Mimus* | *melanotis* | DQ087197 | DQ083931 | AY311576 |
| *Mimus* | *parvulus*_1 | DQ087201 | DQ083935 | AY311553 |
| *Mimus* | *parvulus*_2 | DQ087200 | DQ083934 | AY311553 |
| *Mimus* | *patagonicus* | EF486785 | EF484225 | EF468201 |
| *Mimus* | *polyglottos* | EF486786 | EF484226 | EF468202 |
| *Mimus* | *saturninus* | EF486787 | EF484227 | EF468203 |
| *Mimus* | *thenca* | EF486788 | EF484228 | EF468204 |
| *Mimus* | *trifasciatus* | DQ087195 | DQ083929 | AY311544 |
| *Mimus* | *triurus* | EF486789 | EF484229 | EF468205 |
| *Oreoscoptes* | *montanus* | EF486790 | EF484230 | EF468206 |
| *Ramphocinclus* | *brachyurus* | EF486791 | EF484231 | EF468207 |
| *Toxostoma* | *cinereum* | EF486792 | EF484232 | EF468208 |
| *Toxostoma* | *rufum* | EF486793 | EF484233 | EF468209 |

**Table S5**  **–** Genbank accession numbers used in Galápagos warbler phylogenetic analyses.

| **Genus** | **Species** | **Subspecies** | **Control region** | **ATPase** | **ND2** |
| --- | --- | --- | --- | --- | --- |
| *Dendroica* | *petechia* | *aureola* | JQ218965 | JQ219023 | JQ218947 |
| *Dendroica* | *petechia* | *aureola* | JQ218967 | JQ219024 | JQ218948 |
| *Dendroica* | *petechia* | *cruciana* | JQ218979 | JQ219025 | JQ218949 |
| *Dendroica* | *petechia* | *bartholemica* | JQ218981 | JQ219026 | JQ218950 |
| *Dendroica* | *petechia* | *babad* | JQ218982 | JQ219027 | JQ218951 |
| *Dendroica* | *petechia* | *gundlachi* | JQ218984 | JQ219028 | JQ218952 |
| *Dendroica* | *petechia* | *paraguanae* | JQ218988 | JQ219029 | JQ218953 |
| *Dendroica* | *petechia* | *chrysendeta* | JQ218992 | JQ219030 | JQ218954 |
| *Dendroica* | *petechia* | *xanthotera* | JQ218994 | JQ219031 | JQ218955 |
| *Dendroica* | *petechia* | *aequatorialis* | JQ218997 | JQ219032 | JQ218956 |
| *Dendroica* | *petechia* | *eoa* | JQ219000 | JQ219033 | JQ218957 |
| *Dendroica* | *petechia* | *ruficapilla* | JQ219007 | JQ219034 | JQ218958 |
| *Dendroica* | *petechia* | *aurifrons* | JQ219008 | JQ219035 | JQ218959 |
| *Dendroica* | *petechia* | *melanoptera* | JQ219010 | JQ219036 | JQ218960 |
| *Dendroica* | *petechia* | *aestiva* | JQ219019 | JQ219037 | JQ218961 |
| *Dendroica* | *petechia* | *aestiva* | JQ219022 | JQ219038 | JQ218962 |
| *Dendroica* | *pensylvanica* | *NA* | AF206016 | AY650194 | AY650194 |

**Table S6** **-** Genbank accession numbers used as additional outgroup taxa in the phylogenetic analysis of DF.

| **Genus** | **Species** | **Accession** |
| --- | --- | --- |
| *Tiaris* | *olivaceus* | GU215362 |
| *Tiaris* | *olivaceus* | AF489901 |
| *Tiaris* | *olivaceus* | GU215363 |
| *Tiaris* | *olivaceus* | AF447375 |
| *Tiaris* | *obscura* | AF108808 |
| *Tiaris* | *obscura* | AF108807 |
| *Tiaris* | *canora* | HQ153058 |
| *Tiaris* | *canora* | AF310042 |
| *Tiaris* | *bicolor* | AF489899 |
| *Tiaris* | *bicolor* | AF310044 |
| *Tiaris* | *bicolor* | EF529971 |
| *Tiaris* | *bicolor* | AF290152 |
| *Tiaris* | *bicolor* | AY700048 |
| *Tiaris* | *bicolor* | JF262134 |
| *Melanospiza* | *richardsoni* | AF310043 |
| *Loxipasser* | *anoxanthus* | AF489888 |

**Table S7** - **Models and rates of molecular evolution.** These were employed in the Bayesian divergence-time analysis for each of the loci. Rates are sequence divergence per lineage per million years.

| **Galápagos taxon** | **Clade name** | **Mitochondrial regions** | **Model** | **Alignment length (base pairs)** | **Clock rate (%)** |
| --- | --- | --- | --- | --- | --- |
| Galápagos warbler | Genus *Dendroica* | Control region; ATPase gene; ND2 | GTR+I+G | 2227 | 1.95 (Chaves *et al.* 2012) |
| Galápagos flycatcher | Genus *Myarchus* | Cytochrome B, ND2 | HKY+G | 2010 | 2.07 (Weir & Schluter 2008) |
| Galapagos mockingbirds | Genus *Mimus* | CO1, ND2, ATPase | GTR+I+G | 3582 | 2.07 (Weir & Schluter 2008) |
| Galápagos dove | Genus *Zenaida* | Cytochrome B, ND2 | GTR+G | 2137 | 1.96 (Weir & Schluter 2008) |
| Darwin’s finches | Finches | Cytochrome B | GTR+I+G | 921 | 2.07 (Weir & Schluter 2008) |

**Table S8 – DAISIE models.** Description of the DAISIE models fitted to the Galápagos phylogenetic dataset, specifying the parameters of each model. λ^c^ - per lineage rate of cladogenesis; μ - per lineage rate of extinction; *K*’ – carrying capacity; γ - per lineage rate of immigration; λ^a^ - per lineage rate of anagenesis; DD – diversity-dependence in λ^c^ and γ.

| **Name** | **Background** | | | | |  | **Darwin finch type** | | | **Pars** | **Description** |
| --- | --- | --- | --- | --- | --- | --- | --- | --- | --- | --- | --- |
|  | **λ^c^** | **μ** | ***K*’** | **γ** | **λ^a^** |  | **λ^c^** | **μ** | **K’** |  |  |
| M1 | x | x |  | x | x |  |  |  |  | 4 | Homogenous rates across all lineages. No DD. |
| M2 | x | x |  | x | x |  | x |  |  | 5 | DF-type species have a different cladogenesis rate. No DD. |
| M3 | x | x |  | x | x |  |  | x |  | 5 | DF-type species have a different extinction rate. No DD. |
| M4 | x | x |  | x | x |  |  |  | x | 5 | DD is in operation for DF-types. |
| M5 | x | x |  | x | x |  | x | x |  | 6 | DF-type species have different cladogenesis and extinction rates. No DD. |
| M6 | x | x |  | x | x |  | x |  | x | 6 | DF-types have a different cladogenesis rate and DD. |
| M7 | x | x |  | x | x |  |  | x | x | 6 | DF-types have a different extinction rate and DD. |
| M8 | x | x |  | x | x |  | x | x | x | 7 | DF-types have different cladogenesis and extinction rate and DD. |
| M1’ | x | x | x | x | x |  |  |  |  | 5 | Homogenous rates across all lineages. DD is in operation. |
| M2’ | x | x | x | x | x |  | x |  |  | 6 | DF-type species have a different cladogenesis rate. DD is in operation for non-DF-type species. |
| M3’ | x | x | x | x | x |  |  | x |  | 6 | DF-type species have a different extinction rate. DD is in operation for non-DF-type species. |
| M4’ | x | x | x | x | x |  |  |  | x | 6 | Both types have DD but *K*' varies between DF and non-DF-types. |
| M5’ | x | x | x | x | x |  | x | x |  | 7 | DF-type species differ cladogenesis and extinction rates. DD is in operation for non-DF-type species |
| M6’ | x | x | x | x | x |  | x |  | x | 7 | DD is in operation for both types. DF-type species differ in cladogenesis and *K*'. |
| M7’ | x | x | x | x | x |  |  | x | x | 7 | DD is in operation for both types. DF-type species differ in extinction and *K*'. |
| M8’ | x | x | x | x |  |  | x | x | x | 8 | DD is in operation for both types. DF-type species differ in cladogenesis, extinction and *K*'. |

**Table S9** – **ML values and parameter estimates**. Estimates obtained by fitting the different DAISIE models to the ‘consensus’ Galápagos phylogenetic dataset. Models are described in detail in Table S8. Loglik – Log likelihood; BIC – Bayesian information criterion.

| **Name** | **Background** | | | | |  | **Darwin’s finch type** | | | **Loglik** | **BIC** | **BIC weight** |
| --- | --- | --- | --- | --- | --- | --- | --- | --- | --- | --- | --- | --- |
|  | **λ^c^** | **μ** | ***K*’** | **γ** | **λ^a^** |  | **λ^c^** | **μ** | ***K*’** |  |  |  |
| M1 | 2.55 | 2.68 | - | 0.009 | 1.01 |  | - | - | - | -76.0 | 186.98 | 1.81x10^-1^ |
| M2 | 0.38 | 0.55 | - | 0.004 | 1.10 |  | 2.28 | - | - | -72.3 | 188.36 | 9.14 x10^-2^ |
| M3 | 2.48 | 2.70 | - | 0.009 | 1.02 |  | - | 2.25 | - | -75.7 | 195.17 | 3.03 x10^-3^ |
| M4 | 2.55 | 2.68 | - | 0.009 | 1.01 |  | - | - | ∞ | -76.0 | 195.73 | 2.29 x10^-3^ |
| M5 | 0.29 | 0.38 | - | 0.004 | 1.03 |  | 6.87 | 6.51 | - | -67.7 | 187.81 | 1.20 x10^-1^ |
| M6 | 0.38 | 0.55 | - | 0.004 | 1.10 |  | 2.28 | - | ∞ | -72.3 | 197.10 | 1.15 x10^-3^ |
| M7 | 2.48 | 2.70 | - | 0.009 | 1.02 |  | - | 2.25 | ∞ | -75.7 | 203.92 | 3.83 x10^-5^ |
| M8 | 0.20 | 0.09 | - | 0.002 | 0.87 |  | ≫ μ*K*’ | 8.91 | 14.99 | -61.7 | 184.64 | 5.86 x10^-1^ |
| M1’ | 2.56 | 2.69 | ∞ | 0.009 | 1.01 |  | - | - | - | -76.0 | 195.73 | 2.29 x10^-3^ |
| M2’ | 0.42 | 0.52 | 23.7 | 0.004 | 1.10 |  | 2.27 | - | - | -72.3 | 197.09 | 1.16 x10^-3^ |
| M3’ | 2.65 | 0.46 | 3.7 | 0.003 | 1.32 |  | - | 2.07 | - | -73.4 | 199.29 | 3.87 x10^-4^ |
| M4’ | 1.84 | 0.49 | 4.0 | 0.003 | 1.25 |  | - |  | ∞ | -74.5 | 201.52 | 1.26 x10^-4^ |
| M5’ | 0.33 | 0.36 | 17.3 | 0.004 | 1.02 |  | 6.85 | 6.48 |  | -67.7 | 196.53 | 1.53 x10^-3^ |
| M6’ | 0.42 | 0.52 | 23.7 | 0.004 | 1.10 |  | 2.27 |  | ∞ | -72.3 | 205.83 | 1.47 x10^-5^ |
| M7’ | 2.65 | 0.46 | 3.7 | 0.003 | 1.32 |  |  | 2.07 | ∞ | -73.4 | 208.03 | 4.88 x10^-6^ |
| M8’ | 0.33 | 0.05 | 5.4 | 0.002 | 0.90 |  | ≫ μ*K*’ | 8.91 | 14.99 | -61.6 | 193.18 | 8.20 x10^-3^ |

**Table S10** – **Precision of the M8 model.** Precision of the ML estimates for the M8 model, based on 2000 simulated datasets. λ^c^ - per lineage rate of cladogenesis; μ - per lineage rate of extinction; *K*’ – carrying capacity; γ - per lineage rate of immigration; λ^a^ - per lineage rate of anagenesis.

| **Parameter** | **Simulated value** | **Estimated values (percentiles)** | | |
| --- | --- | --- | --- | --- |
|  |  | **25%** | **50%** | **75%** |
| λ^c^ | 0.20 | 0.09 | 0.18 | 0.28 |
| μ | 0.09 | 0 | 0.16 | 0.38 |
| γ | 0.002 | 0.0020 | 0.0027 | 0.003 |
| λ^a^ | 0.87 | 0.55 | 0.94 | 2.04 |
| λ^c^_DF-type_ | 3755 | 3917 | 7975 | 1.3 x 10^10^ |
| μ _DF-type_ | 8.91 | 7.99 | 9.26 | 10.67 |
| *K’*_DF-type_ | 14.9 | 14.74 | 14.99 | 15 |

**Table S11**  – **95% confidence intervals of the ML parameter estimates for the three best models across the posterior distribution of phylogenetic datasets**. Obtained by fitting DAISIE models across 100 datasets incorporating phylogenetic uncertainty. Models are described in detail in Table S8.

| **Model** | **Background** | | | |  | **Darwin’s finch type** | | |
| --- | --- | --- | --- | --- | --- | --- | --- | --- |
|  | **λ^c^** | **μ** | **γ** | **λ^a^** |  | **λ^c^** | **μ** | ***K*’** |
| M1 | 1.78 – 3.24 | 1.82 – 3.42 | 0.007 – 0.011 | 0.14 – 1.88 |  | - | - | - |
| M5 | 0.23 – 0.36 | 0.26 – 0.56 | 0.003 – 0.004 | 0.85 – 1.28 |  | 3.58 – 9.99 | 3.22 – 9.71 | - |
| M8 | 0.16 – 0.24 | 0.01 – 0.21 | 0.002 – 0.0025 | 0.76 – 1.08 |  | 1x10^3^ – Inf | 4.88 – 11.50 | 14-15 |

**Table S12** – ML parameters of the best model for different assumed archipelago ages. λ^c^ - per lineage rate of cladogenesis; μ - per lineage rate of extinction; *K*’ – carrying capacity; γ - per lineage rate of immigration; λ^a^ - per lineage rate of anagenesis.

|  |  | **Background** | | | |  | **DF-type** | | |  |  |
| --- | --- | --- | --- | --- | --- | --- | --- | --- | --- | --- | --- |
| **Assumed archipelago age (My)** | **Best model** | **λ^c^** | **μ** | **γ** | **λ^a^** |  | **λ^c^** | **μ** | ***K’*** |  | **Loglik** |
| 4 | M8 | 0.2 | 0.09 | 0.002 | 0.87 |  | ≫ μ*K*’ | 8.91 | 15 |  | -61.71 |
| 5 | M8 | 0.23 | 0.24 | 0.002 | 0.93 |  | ≫ μ*K*’ | 8.86 | 15 |  | -63.18 |
| 6 | M1 | 2.69 | 2.95 | 0.01 | 0.96 |  | - | - | - |  | -76.77 |
| 7 | M1 | 2.72 | 3.02 | 0.01 | 0.94 |  | - | - | - |  | -76.94 |
| 8 | M1 | 2.74 | 3.06 | 0.01 | 0.93 |  | - | - | - |  | -77.05 |
| 9 | M1 | 2.75 | 3.10 | 0.01 | 0.93 |  | - | - | - |  | -77.12 |
| 10 | M1 | 2.76 | 3.12 | 0.01 | 0.92 |  | - | - | - |  | -77.16 |
| 11 | M1 | 2.76 | 3.13 | 0.01 | 0.92 |  | - | - | - |  | -77.19 |
| 12 | M1 | 2.76 | 3.14 | 0.01 | 0.92 |  | - | - | - |  | -77.21 |
| 13 | M1 | 2.77 | 3.15 | 0.01 | 0.92 |  | - | - | - |  | -77.22 |
| 14 | M1 | 2.77 | 3.15 | 0.01 | 0.92 |  | - | - | - |  | -77.23 |
| 15 | M1 | 2.77 | 3.16 | 0.01 | 0.92 |  | - | - | - |  | -77.23 |

**Table S13**  – ML parameters of the M8 model for different *P*_DF-type_ values. λ^c^ - per lineage rate of cladogenesis; μ - per lineage rate of extinction; *K*’ – carrying capacity; γ - per lineage rate of immigration; λ^a^ - per lineage rate of anagenesis.

|  | **Background** | | | |  | **DF-type** | | |  |  |
| --- | --- | --- | --- | --- | --- | --- | --- | --- | --- | --- |
| ***P*_DF-type_** | **λ^c^** | **μ** | **γ** | **λ^a^** |  | **λ^c^** | **μ** | ***K’*** |  | **Loglik** |
| 0.001 | 0.32 | 0.48 | 0.004 | 1.07 |  | ≫ μ*K*’ | 8.96 | 14.01 |  | -61.11 |
| 0.01 | 0.31 | 0.44 | 0.004 | 1.05 |  | ≫ μ*K*’ | 8.96 | 15.00 |  | -61.18 |
| 0.1 | 0.23 | 0.21 | 0.003 | 0.94 |  | ≫ μ*K*’ | 8.91 | 14.04 |  | -61.59 |
| 0.11 | 0.22 | 0.19 | 0.003 | 0.93 |  | ≫ μ*K*’ | 8.96 | 14.79 |  | -61.61 |
| 0.12 | 0.22 | 0.17 | 0.002 | 0.92 |  | ≫ μ*K*’ | 8.96 | 14.79 |  | -61.64 |
| 0.13 | 0.21 | 0.15 | 0.002 | 0.91 |  | ≫ μ*K*’ | 8.96 | 14.95 |  | -61.66 |
| 0.14 | 0.21 | 0.13 | 0.002 | 0.90 |  | ≫ μ*K*’ | 8.96 | 14.91 |  | -61.68 |
| 0.15 | 0.20 | 0.11 | 0.002 | 0.89 |  | ≫ μ*K*’ | 8.96 | 14.93 |  | -61.69 |
| 0.16 | 0.20 | 0.09 | 0.002 | 0.88 |  | ≫ μ*K*’ | 8.96 | 14.95 |  | -61.71 |
| 0.17 | 0.19 | 0.07 | 0.002 | 0.87 |  | ≫ μ*K*’ | 8.96 | 14.89 |  | -61.72 |
| 0.18 | 0.19 | 0.06 | 0.002 | 0.86 |  | ≫ μ*K*’ | 8.96 | 14.85 |  | -61.73 |
| 0.19 | 0.18 | 0.04 | 0.002 | 0.85 |  | ≫ μ*K*’ | 8.96 | 14.80 |  | -61.73 |
| 0.2 | 0.18 | 0.02 | 0.002 | 0.84 |  | ≫ μ*K*’ | 8.96 | 14.86 |  | -61.74 |
| 0.21 | 0.17 | 0.01 | 0.002 | 0.83 |  | ≫ μ*K*’ | 8.96 | 14.93 |  | -61.74 |
| 0.22 | 0.17 | 0.00 | 0.002 | 0.83 |  | ≫ μ*K*’ | 8.96 | 14.55 |  | -61.74 |
| 0.23 | 0.17 | 0.00 | 0.002 | 0.83 |  | ≫ μ*K*’ | 8.96 | 14.64 |  | -61.74 |
| 0.24 | 0.17 | 0.00 | 0.002 | 0.83 |  | ≫ μ*K*’ | 8.96 | 14.64 |  | -61.74 |
| 0.25 | 0.17 | 0.00 | 0.002 | 0.83 |  | ≫ μ*K*’ | 8.96 | 14.64 |  | -61.74 |
| 0.28 | 0.17 | 0.00 | 0.002 | 0.83 |  | ≫ μ*K*’ | 8.91 | 15.00 |  | -61.74 |

**Table S14 -** ML parameters of the M8 model for different *Progne modesta* maximum colonisation times (My). λ^c^ - per lineage rate of cladogenesis; μ - per lineage rate of extinction; *K*’ – carrying capacity; γ - per lineage rate of immigration; λ^a^ - per lineage rate of anagenesis. *Value used in the main analyses.

|  | **Background** | | | |  | **DF-type** | | |  |  |
| --- | --- | --- | --- | --- | --- | --- | --- | --- | --- | --- |
| **Colonisation Time** | **λ^c^** | **Μ** | **γ** | **λ^a^** |  | **λ^c^** | **μ** | ***K’*** |  | **Loglik** |
| 0.01 | 0.20 | 1.0E-01 | 0.0023 | 0.91 |  | ≫ μ*K*’ | 8.91 | 15.0 |  | -63.81 |
| 0.086* | 0.20 | 8.7E-02 | 0.0022 | 0.88 |  | ≫ μ*K*’ | 8.91 | 14.1 |  | -61.71 |
| 0.1 | 0.19 | 8.4E-02 | 0.0022 | 0.87 |  | ≫ μ*K*’ | 8.91 | 15.0 |  | -61.57 |
| 0.5 | 0.17 | 2.2E-02 | 0.0021 | 0.76 |  | ≫ μ*K*’ | 8.91 | 15.0 |  | -60.20 |
| 1 | 0.16 | 2.1E-12 | 0.0020 | 0.69 |  | ≫ μ*K*’ | 8.91 | 15.0 |  | -59.76 |
| 2 | 0.15 | 1.2E-10 | 0.0020 | 0.61 |  | ≫ μ*K*’ | 8.89 | 15.0 |  | -59.49 |
| 3 | 0.14 | 2.3E-10 | 0.0020 | 0.56 |  | ≫ μ*K*’ | 8.91 | 15.0 |  | -59.46 |
| 4 | 0.14 | 4.6E-11 | 0.0020 | 0.52 |  | ≫ μ*K*’ | 8.91 | 15.0 |  | -59.50 |

**Supporting Information References**

1.

Drummond, A.J. & Rambaut, A. (2007). BEAST: Bayesian evolutionary analysis by sampling trees. *BMC Evol. Biol.*, 7, 214.

2.

Farrington, H.L., Lawson, L.P., Clark, C.M. & Petren, K. (2014). The evolutionary history of Darwin’s finches: speciation, gene flow, and introgression in a fragmented landscape. *Evolution*.

3.

Grant, P.R. & Grant, B.R. (2008). *How and Why Species Multiply: The Radiation of Darwin’s Finches*. Princeton University Press, Princeton.

4.

Jetz, W., Thomas, G.H., Joy, J.B., Hartmann, K. & Mooers, A.O. (2012). The global diversity of birds in space and time. *Nature*, 491, 444–8.

5.

Steadman, D.W., Stafford, T.W., Donahue, D.J. & Jull, A.J.T. (1991). Chronology of Holocene vertebrate extinction in the Galápagos Islands. *Quat. Res.*, 36, 126–133.

6.

Weir, J.T. & Schluter, D. (2008). Calibrating the avian molecular clock. *Mol. Ecol.*, 17, 2321–2328.
